# Precise Generation of Human Induced Pluripotent Stem Cell–derived Cell Lines Harboring Disease-relevant Single Nucleotide Variants Using a Prime Editing System

**DOI:** 10.21769/BioProtoc.5191

**Published:** 2025-02-20

**Authors:** Seiya Kanno, Kota Sato, Toru Nakazawa

**Affiliations:** 1 Department of Ophthalmology, Tohoku University Graduate School of Medicine, Sendai, Japan; 2 Department of Advanced Ophthalmic Medicine, Tohoku University Graduate School of Medicine, Sendai, Japan; 3 Department of Ophthalmic Imaging and Information Analytics, Tohoku University Graduate School of Medicine, Sendai, Japan; 4 Department of Retinal Disease Control, Tohoku University Graduate School of Medicine, Miyagi, Japan

**Keywords:** Human induced pluripotent stem (iPS) cell, Prime editing, CRISPR/Cas9, Precision genome editing, pegRNA design, Disease-associated single nucleotide variant (SNV), Disease modeling, Isogenic cell line

## Abstract

Human induced pluripotent stem (iPS) cell lines harboring mutations in disease-related genes serve as invaluable in vitro models for unraveling disease mechanisms and accelerating drug discovery efforts. Introducing mutations into iPS cells using traditional gene editing approaches based on the CRISPR-Cas9 endonuclease often encounters challenges such as unintended insertions/deletions (indels) and off-target effects. To address these limitations, we present a streamlined protocol for introducing highly accurate gene mutations into human iPS cells using prime editing, a “search-and-replace” genome-editing technology that combines unwanted indel-minimized CRISPR-Cas9 nickase with reverse transcriptase. This protocol encompasses the design of prime editing guide RNAs (pegRNAs) required for binding and replacement at target loci, construction of prime editor and pegRNA expression vectors, gene transfer into iPS cells, and cell line selection. This protocol allows for the efficient establishment of disease-associated gene variants within 6–8 weeks while preserving critical genomic context.

Key features

• Dramatic improvement in efficiency of In-Fusion cloning using inserts assembled from the three pegRNA components (spacer, spCas9 scaffold, and 3' extension) via overlap extension PCR.

• Cost-effective and time-saving selection of pegRNAs for prime editing via bulk Sanger sequencing.

• Straightforward gene transfection using polymer-based reagents, which requires no specialized equipment or techniques and offers high reproducibility and broad applicability across different cell lines.

• Precise genome editing based on pegRNA/prime editing minimizes off-target effects, enabling a wide range of applications in the study of disease-associated genetic variants.

Graphical overview

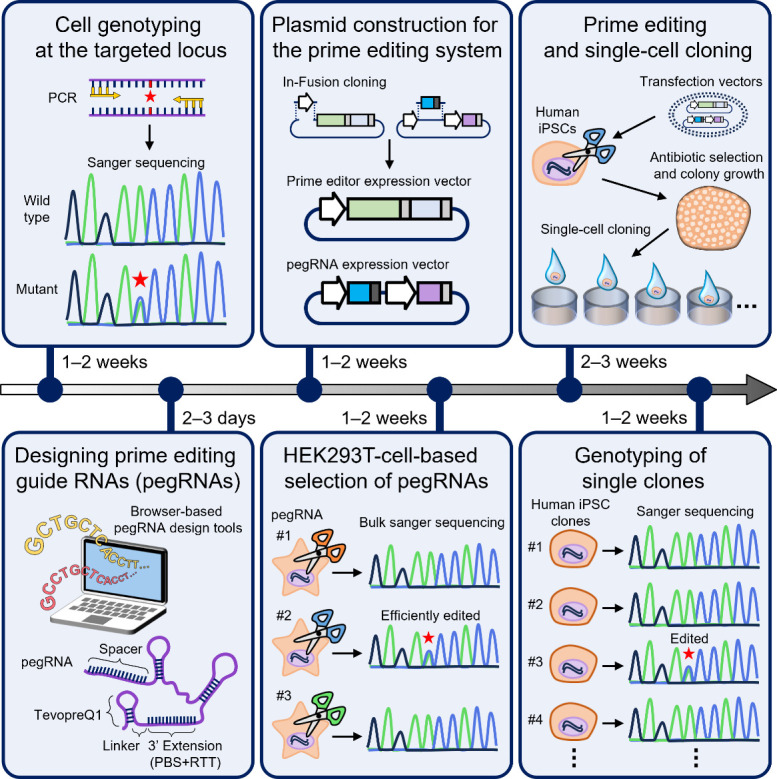

Key steps of generation of human induced pluripotent stem (iPS) cell lines harboring disease-relevant single nucleotide variants (SNVs) using a prime editing system

## Background

Advances in genomic disease analysis have identified numerous single nucleotide variants (SNVs) associated with diverse diseases. Patient-derived human induced pluripotent stem (iPS) cells carrying these SNVs offer valuable in vitro models for elucidating disease mechanisms and accelerating drug discovery. However, phenotypic variability arising from diverse genetic backgrounds among iPS cell lines can confound the accurate assessment of disease gene effects [1].

Recent advancements in genome editing technologies have enabled the establishment of highly improved protocols for generating isogenic iPS cell lines through homology-directed repair (HDR)-mediated SNVs editing using CRISPR-Cas9 and single-stranded oligodeoxynucleotides (ssODNs), facilitating functional analyses of SNVs [2–5]. Nonetheless, these methods often introduce unintended insertions or deletions (indels) and off-target effects due to the induction of double-strand breaks in the DNA by CRISPR-Cas9, limiting their precision and efficiency [6].

To address these limitations, we present a protocol for generating iPS cell lines with precisely engineered SNVs in genes of interest using prime editing. Prime editing is a “search-and-replace” genome editing tool that combines a modified CRISPR-Cas9 nickase with reverse transcriptase to minimize unwanted indels [6]. This approach enables the functional analysis of gene variants while preserving the genomic context, providing a more controlled and informative system for studying disease mechanisms. Specifically, this protocol presents a workflow for generating a cell line harboring the *OPTN* p.(Asn51Thr) missense mutation, which was previously identified in our laboratory as an SNV associated with normal-tension glaucoma (NTG) [7].

This protocol has several key steps. First, the necessary components for the prime editing system are prepared, including the prime editor enzyme and prime editing guide RNA (pegRNA) expression vectors designed to introduce specific mutations. Additionally, a selectable marker gene, such as the puromycin resistance gene, is included to identify and select cells that have successfully taken up the editing components. Next, each pegRNA is introduced into HEK293T cells along with the prime editor enzyme. To assess the efficiency of the designed pegRNAs, the sequence of the target loci is analyzed in a bulk population of transfected cells. Finally, the pegRNA and prime editor enzyme are introduced into a human iPS cell line, 201B7, and individual iPS cell clones are isolated from the transfected population. By focusing on pegRNAs with high editing efficiency, the chances of obtaining iPS cell lines with the desired gene mutations are increased.

This protocol offers a robust and efficient method for generating iPS cell lines with targeted gene mutations, not only for studying disease-related genes but also for correcting pathogenic variants in patient-derived cells. While prime editing offers significant advantages over traditional methods, it is not without its limitations. For example, although several efficient pegRNA design tools are available, more information is needed in the future regarding editing efficiency in human iPS cells, as it can vary depending on the target locus and cell type. Furthermore, experimental conditions, such as reagent concentrations, transfection methods, and selection markers, must be carefully optimized for each iPS cell line to achieve optimal results.

## Materials and reagents

**Biological materials**

1. Competent quick DH5α (Toyobo, catalog number: DNA-913F); store in a -80 °C ultra-low temperature freezer

2. Human iPS cell line 201B7 (RIKEN BRC Cell Bank, catalog number: HPS0063); to prevent a decrease in cell viability, iPS cells should be immediately transferred to liquid nitrogen for storage after receiving them from the supplier or after creating a cell bank

3. pLV[Exp]-EF1A>hCas9(ns):T2A:Puro (VectorBuilder, catalog number: VB210412-1054sbc)

4. pCMV-PEmax-P2A-hMLH1dn (Addgene, catalog number: 174828)

5. pU6-pegRNA-GG-acceptor (Addgene, catalog number: 132777)

6. pMK232 (CMV-OsTIR1-PURO) (Addgene, catalog number: 72834)

Store plasmids in a freezer at -30 °C.

**Reagents**

1. StemFit medium (Ajinomoto Healthy Supply, catalog number: AK03N)

2. DMEM (4.5 g/L glucose) with L-Gln and sodium pyruvate (Nacalai Tesque, catalog number: 08458-45)

3. Fetal bovine serum (FBS) (Thermo Fisher Scientific, catalog number: 12483020)

4. DPBS, no calcium, no magnesium (Thermo Fisher Scientific, catalog number: 14190144)

5. Trypsin-EDTA (0.25%), phenol red (Thermo Fisher Scientific, catalog number: 25200056); dilute with DPBS as needed.

6. TrypLE Select enzyme (1×), no phenol red (Thermo Fisher Scientific, catalog number: 12563011)

7. Penicillin-streptomycin (Thermo Fisher Scientific, catalog number: 15140122)

8. CultureSure Y-27632 (Fujifilm Wako Pure Chemical, catalog number: 036-24023)

9. iMatrix-511 (Matrixome, catalog number: 892012)

10. STEM-CELLBANKER GMP grade (Zenogen Pharma, catalog number: 11924)

11. PolyJet DNA in vitro transfection reagent (SignaGen Laboratories, catalog number: SL100688)

12. Puromycin (InvivoGen, catalog number: ant-pr)

13. QIAamp DNA Mini kit (Qiagen, catalog number: 51306)

14. NucleoSpin gel and PCR clean-up (Macherey-Nagel, catalog number: 740609.250)

15. SOC medium (Thermo Fisher Scientific, catalog number: 15544034)

16. Plasmid Miniprep Plus Purification kit (BioElegen Technology, catalog number: DP01-PLUS-300)

17. KOD One PCR Master Mix (Toyobo, catalog number: KMM-101)

18. Dpn I (New England Biolabs, catalog number: R0176L)

19. In-fusion snap assembly master mix (Takara Bio, catalog number: 638947)

20. Ampicillin sodium (Fujifilm Wako Pure Chemical, catalog number: 016-23301)

21. BD Difco LB (Luria-Bertani) broth Miller (Becton, Dickinson and Company, catalog number: 244620)

22. BD BACTO agar (Becton, Dickinson and Company, catalog number: 214010)

23. BD Difco 2×YT (yeast extract tryptone medium) (Becton, Dickinson and Company, catalog number: 244020)

24. Agarose S (Nippon Gene, catalog number: 318-01195)

25. Gel loading dye, purple (6×), no SDS (New England Biolabs, catalog number: B7025S)

26. 1 kb DNA ladder (Nippon Genetics, catalog number: NE-MWD1P)

27. 100 bp DNA ladder (Takara Bio, catalog number: 3422D)

28. Primers (OPC cartridge purified from Eurofins Genomics)

Homologous sequences for overlap PCR or in-fusion cloning are displayed in lowercase:

OPTN_genotyping_forward: 5'- AATCGCCAATGGGTTTGTGGGAC -3'

OPTN_genotyping_reverse: 5'- TGCTAAATCCTGTGCTTCCCCACC -3'

EF1A_ forward: 5'- ccagatatacgcgttGGCTCCGGTGCCCGTCAGTG -3'

EF1A_reverse: 5'- acggttcactaaaccTCACGACACCTGAAATG -3'

Inverse_PEmax_forward: 5'- ggtttagtgaaccgtCAGATCCGC -3'

Inverse_PEmax_reverse: 5'- aacgcgtatatctggCCCGTACATC -3'

EF1A_sequence_forward1: 5'- GGTGGAGACTGAAGTTAGGCCAGC -3'

EF1A_sequence_forward2: 5'- TAAGTGCAGTAGTCGCCGTGAACG -3'

EF1A_sequence_reverse: 5'- CACGCAAGGGCCATAACCCG -3'

tevopreQ1 + Terminator_forward: 5'- CGCGGTTCTATCTAGTTACGCGTTAAACCAACTAGAA -3'

tevopreQ1 + Terminator_reverse: 5'- AAAAAATTCTAGTTGGTTTAACGCGTAACTAGATAGAACCGCG -3'

Spacer_OPTN_forward: 5'- gaaaggacgaaacaccGCTGCTCACCTTTCAGCTGG -3'

Spacer_OPTN_reverse: 5'- ttctagctctaaaacCCAGCTGAAAGGTGAGCAGC -3'

spCas9_scaffold_forward: 5'- GTTTTAGAGCTAGAAATAGCAAGTTAAAATAAGGCTAGTCCGTTATCAACTTGA -3'

spCas9_scaffold_reverse: 5'- GCACCGACTCGGTGCCACTTTTTCAAGTTGATAACGGACTAGCCTTATTTTAACTTGC -3'

3’extension + Linker_OPTN_forward: 5'- gcaccgagtcggtgcTGACCGAGACCCACCAGCTGAAAGGACCC -3'

3’extension + Linker_OPTN_reverse: 5'- ctagatagaaccgcgATTAGGGTCCTTTCAGCTGGTGGGTCTCGGTC -3'

Inverse_U6-pegRNA_Forward: 5'- cgcggttctatctagTTACGCG -3'

Inverse_U6-pegRNA_reverse: 5'- ggtgtttcgtcctttcCACAAGA -3'

pegRNA_sequence_forward: 5'- GAGGGCCTATTTCCCATGATTCC -3'

**Laboratory supplies**

1. 15 mL centrifuge tubes (TPP, catalog number: 91015)

2. 1.5 mL microcentrifuge tubes (Ina-optika, catalog number: 113004)

3. 0.5 mL microcentrifuge tube (Ina-optika, catalog number: SC-005)

4. 20 μL racked tips, sterile (Eppendorf, catalog number: 0030075226)

5. 200 μL racked tips, sterile (Eppendorf, catalog number: 0030075234)

6. 1000 μL racked tips, sterile (BM Equipment, catalog number: WE1000-RL)

7. 12-well cell culture plate (Corning, catalog number: 353043)

8. 24-well cell culture plate (Corning, catalog number: 353047)

9. 48-well cell culture plate (Corning, catalog number: 353078)

10. 96-well cell culture plate (Corning, catalog number: 353072)

11. 60 × 15 mm cell culture dish (Corning, catalog number: 353004)

12. STAR SDish9015 ver. 2 Petri dish (Rikaken, catalog number: RSU-SD9015-2)

13. 0.2 mL 8-tube PCR strips (Bio-Rad Laboratories, catalog number: TLS0801)

14. 0.2 mL domed PCR tube 8-cap strips (Bio-Rad Laboratories, catalog number: TCS0801)

15. 2 mL cryo vial, inner screw (Simport Scientific, catalog number: T311-2)

16. BICELL freezing treatment container (Nihon Freezer, catalog number: BICELL)

## Equipment

1. Thermal cycler (Bio-Rad, model: C1000 Touch)

2. Refrigerated centrifuge (Tomy Seiko, model: LX-120)

3. High-speed refrigerated microcentrifuge (Tomy Seiko, model: MX300)

4. Cell culture incubator (PHC, catalog number: MCO-170AICUVD-PJ)

5. Shaker incubator (Taitec, model: BR-40LF)

6. Benchtop incubator for bacterial culture (AS ONE, model: PIC-100)

7. Microvolume spectrophotometer (Thermo Fisher Scientific, model: NanoDrop 2000c)

8. Gel electrophoresis equipment (Takara Bio, model: Mupid-exU)

9. Gel imaging system (Bio-Rad, model: GelDoc Go imaging system)

10. Autoclave (Tomy Seiko, model: LBS-245)

## Software and datasets

1. SnapGene Viewer (SnapGene, https://www.snapgene.com/snapgene-viewer)

## Procedure

**A. Identification of the genotype at the targeted SNV locus in the cell line**

As the first step, it is necessary to investigate the impact of the target gene mutation in advance, as the effort required to establish cell lines differs depending on whether heterozygous or homozygous cell lines are ultimately needed after editing. Databases such as ClinVar (https://www.ncbi.nlm.nih.gov/clinvar/) and gnomAD (https://gnomad.broadinstitute.org/) can be used to search for information about the target mutation and reports from other researchers. For example, missense mutations like *OPTN* p.(Glu50Lys), which cause early-onset familial NTG, often exhibit pathogenicity even in a heterozygous state in autosomal-dominant genetic diseases. Additionally, mutations with haploinsufficiency or a dominant negative effect are also likely to be pathogenic in a heterozygous state, so depending on the research purpose, a heterozygous cell line may be sufficient.

Next, it is necessary to identify the genotype at the target disease-related locus in the cell line to be used. If the genotype of the cell line to be used is heterozygous, pegRNAs that convert the wild type and mutant types to each other (wild type to mutant type or vice versa) are designed, and prime editing is used with each pegRNA in separate experiments to obtain homozygous cell lines of both the wild type and mutant types. On the other hand, if starting from a homozygous genotype, it is necessary to increase the number of single clones to be obtained in section E, as the frequency of obtaining homozygous cell lines after editing is lower than that of heterozygous cell lines. Alternatively, it is possible to obtain homozygous cell lines by performing editing again after obtaining a heterozygous cell line in a single editing process.

1. Retrieve the genomic sequence surrounding the variant of interest.

a. Search for the gene of interest (e.g., *OPTN*) in the gnomAD database (https://gnomad.broadinstitute.org/).

b. Locate the variant ID of the target variant. If unavailable, use a nearby variant’s ID (e.g., *OPTN* p.Glu50Lys for *OPTN* p.Asn51Thr; not listed).

c. Follow the UCSC link under “External Resources” to visualize the genomic region surrounding the variant.

d. Download the genomic sequence in FASTA format from the “DNA sequence” link under the *View* menu in UCSC.

e. Store the downloaded sequence in a sequence analysis tool such as SnapGene Viewer.

2. Design genotyping primers.

a. Paste the target locus nucleotide sequence into the *PCR template* box of Primer-BLAST (https://www.ncbi.nlm.nih.gov/tools/primer-blast/).

b. Set primer design parameters: amplicon length of 500–700 bp, Tm of 62 °C ± 3 °C, GC content of 50%–80%, and a GC clamp of 1. Click the *Get Primers* button to search for potential primer pairs.

c. Order primers from a qualified supplier (e.g., Eurofins Genomics).

*Note 1: When ordering from Eurofins Genomics, order a 10-nmol-scale oligonucleotide OPC cartridge purified and dissolved in a TE buffer. Upon arrival, store at -20 °C and thaw immediately before use.*

*Note 2: It is recommended to order at least three pairs of primers and conduct PCR experiments to identify the optimal primer pair that specifically amplifies the target gene without any off-target amplification.*

3. Extract and quantify genomic DNA.

a. Extract genomic DNA from the cell line following the manufacturer’s instructions for the genomic DNA extraction kit (e.g., QIAamp DNA Mini Kit).

b. Quantify the extracted DNA concentration using a NanoDrop spectrophotometer at A260.

c. Store the DNA at -20 °C for up to 6 months.

4. Perform PCR with primers spanning the target to amplify and enrich the genomic region of interest.

a. Prepare a 20 µL PCR reaction mixture for each sample (Table 1).

To amplify the region of the *OPTN* p.(Asn51Thr) missense mutation, perform PCR using OPTN_genotyping_forward and OPTN_genotyping_reverse primers.

**Table 1.** PCR reaction mixture components

Reagent	Final concentration	Volume
KOD One PCR master mix (dye-free 2×PCR master mix)	1×	10 μL
Forward primer (2 μM)	0.3 μM	3 μL
Reverse primer (2 μM)	0.3 μM	3 μL
Genomic DNA (20 ng/µL)	4 ng/µL	4 μL
Total	n/a	20 μL

b. Perform two-step PCR using a thermal cycler under the temperature conditions provided in Table 2.

**Table 2.** Thermocycling conditions for PCR

Step	Temp. (°C)	Duration	No. of cycles
Initial denaturation	98	30 s	1
Denaturation	98	10 s	35
Extension	68	5 s/kb	
Hold	4	∞	-

c. Apply 1–2 μL of the PCR product to a 1.5% agarose gel containing a fluorescent nucleic acid stain (e.g., GelRed) and run electrophoresis at 100 V for approximately 15 min.

d. Visualize DNA bands on a transilluminator and verify that the PCR amplicon has migrated to the expected band size.

*Note: Consider using touchdown PCR or nested PCR when you need to increase DNA yield or specificity, such as when amplifying low-abundance targets or dealing with non-specific amplification.*

e. Purify the target amplicon DNA from PCR products using a commercial DNA purification kit (e.g., NucleoSpin Gel and PCR clean-up).

*Note: For single bands, direct purification of DNA from the post-PCR mixture is possible. If extra bands are present, indicating non-specific amplification, excise the desired-sized band from the gel after electrophoresis and extract DNA from the gel.*

f. Quantify the extracted DNA concentration using a NanoDrop spectrophotometer at A260. Store the DNA at -20 °C for up to 6 months.

**Figure 1.**
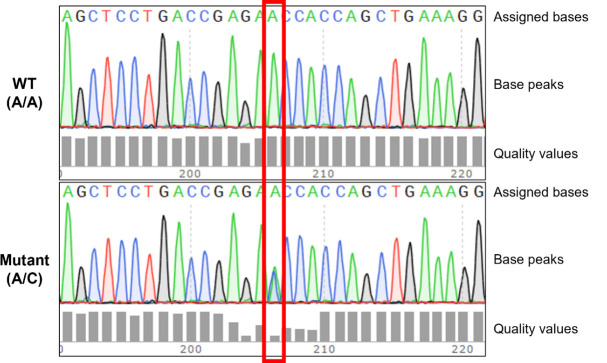
Identification of genotype by analyzing Sanger sequencing waveforms. The red box indicates the position of the *OPTN* p.(Asn51Thr) missense mutation. In cell lines with the wild-type genotype, this position exhibits a homozygous C/C genotype. In cell lines harboring the *OPTN* p.(Asn51Thr) missense mutation, this position displays either a homozygous A/A or a heterozygous C/A genotype. Heterozygosity for C/A is characterized by overlapping peaks of the two bases, often accompanied by lower-quality values.

5. Identify the genotype at the target disease-related locus in the cell line.

a. Send the PCR product to a sequencing service provider (e.g., Eurofins Genomics) for Sanger sequencing to determine the nucleotide sequence. PCR primers within 100–600 bp of the target locus can often serve as sequencing primers. Use OPTN_genotyping_forward as a sequencing primer for the region of the *OPTN* p.(Asn51Thr) missense mutant.

b. Input the nucleotide sequence obtained from Sanger sequencing into Standard Nucleotide BLAST (https://blast.ncbi.nlm.nih.gov/Blast.cgi?PROGRAM=blastn&BLAST_SPEC=GeoBlast&PAGE_TYPE=BlastSearch) to confirm the genotype of the cell line.

c. To account for potential heterozygosity, examine not only the FASTA sequence obtained from Sanger sequencing but also the waveform data. Check whether there are overlapping peaks of two different bases at the same position in the waveform data (Figure 1).

**B. Design of pegRNAs**

PegRNAs are a pivotal component of the prime editing system, directing the targeted insertion of new DNA sequences into a specific genomic locus. A pegRNA consists of three primary domains: a spacer, a reverse transcriptase template (RTT), and a primer binding site (PBS). The spacer, in a complex with the Cas9 protein, recognizes and binds to the target DNA sequence, specifying the precise location for editing. The RTT serves as a template for reverse transcriptase, encoding the new DNA sequence to be inserted. The PBS determines the starting point for reverse transcription, ensuring accurate editing.

Engineered pegRNA (epegRNA), incorporating a trimmed, modified preQ1 riboswitch aptamer (tevopreQ1) at its 3' end, has become prevalent. The stable stem-loop structure formed by tevopreQ1 shields the epegRNA from degradation by cellular RNases, enhancing its stability and, consequently, prime editing efficiency [8]. A linker sequence is inserted between tevopreQ1 and the PBS to prevent unintended base pairing interactions.

In this step, the spacer, RTT, and PBS will be designed for the pegRNA. The design of pegRNA significantly influences both editing efficiency and specificity. Factors such as spacer length, RTT sequence, and PBS position must be optimized. To facilitate efficient design, various browser-based tools, including DeepPrime, pegFinder, and PE-Designer, are employed to generate optimal spacer, RTT, and PBS sequences. DeepPrime, for instance, leverages machine learning to design highly efficient pegRNAs with minimal off-target effects. As the next step of pegRNA design, an appropriate linker sequence is determined to avoid base pairing with other domains of the pegRNA and to ensure optimal reverse transcription. An RNA secondary structure prediction tool, pegLIT, is used for linker design.

1. Use pegRNA design tools such as DeepPrime (https://deepcrispr.info/DeepPrime/), pegFinder (http://pegfinder.sidichenlab.org/), and PE-Designer (http://www.rgenome.net/pe-designer/) to input the target genomic sequence and generate candidate spacer, RTT, and PBS sequences. These tools use machine learning or algorithmic approaches to design optimal pegRNAs.

*Note 1: To optimize prime editing efficiency, it is recommended to pair an 11 nt PBS with an RTT of 12 nt or less, and a 12 nt PBS with an RTT of 13 nt or more [9].*

*Note 2: Regularly check for updates to pegRNA design tools, as features and functionalities may evolve.*

2. Use RNA secondary structure prediction tools like pegLIT (https://peglit.liugroup.us/) to design optimal linker sequences based on the spacer, RTT, and PBS sequences.

3. Order custom-synthesized single-stranded oligonucleotides corresponding to the top and bottom strands of the spacer, 3' extension, linker, and tevopreQ1 sequences from a qualified supplier (e.g., Eurofins Genomics).

**Critical:** To initiate transcription from the RNA polymerase III–dependent U6 promoter, add a guanine or adenine nucleotide to the 5' end of the guide RNA sequence [10].

**Figure 2.**
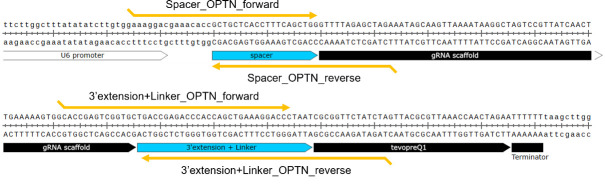
Designed guide RNA for introducing the p.(Asn51Thr) point mutation into the *OPTN* gene using prime editing. PCR primers targeting the spacer and 3' extension + linker are flanked by 15 bp or longer homology regions to enable efficient assembly by overlap PCR or in-fusion cloning.

**Critical:** Order the oligonucleotides so that the 15 bases at the end of each DNA fragment [spacer, scaffold, 3' extension sequence (PBS + RTT + Linker), and backbone vector] are homologous to each other to allow for overlap PCR and in-fusion cloning (**Figure 2**). While the synthesis may be more costly and complex, DNA fragments spanning the spacer-linker region of each designed pegRNA can alternatively be used as inserts for in-fusion cloning.

*Note 1: It is recommended to order at least three distinct pegRNA sequences and comparing their actual editing efficiencies to identify the most effective candidate.*

*Note 2: When ordering from Eurofins Genomics, order a 10-nmol-scale oligonucleotide OPC cartridge purified and dissolved in TE buffer. Upon arrival, store at -20 °C and thaw immediately before use.*

**C. Preparation of constructs for prime editing**

Prime editing (PE) systems have undergone significant advancements in recent years. Initial PE1 systems, which fused SpCas9 with Moloney murine leukemia virus (M-MLV) reverse transcriptase (RT), exhibited limited genome editing efficiency in human cells [11]. PE2 used engineered RT, while PE3 further improved efficiency by introducing a nick into the complementary strand using an additional sgRNA [6]. PEmax is a prime editor protein with optimized codons for both RT and Cas9. PE4 and PE5 transiently inhibited DNA mismatch repair (MMR) by using a dominant-negative mutant of MLH1, a key factor in the MMR pathway, thereby improving editing efficiency. The combination of these systems in PE4Max and PE5Max achieved even higher editing efficiencies [12]. Additionally, the PE7 system improved the stability and integrity of the pegRNA by fusing the N-terminal domain of the RNA-binding protein La to the prime editor protein, thereby enhancing editing efficiency. [13].

**Figure 3.**
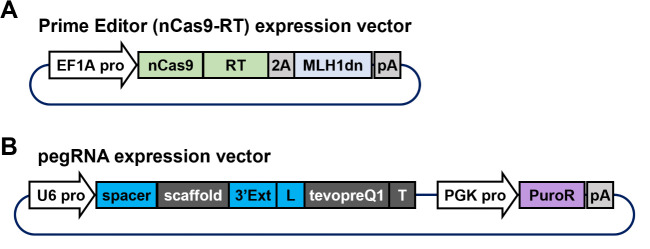
Components of the plasmid vector required for the prime editing system. A. A plasmid vector co-expressing prime editor and MLH1dn under the EF1A promoter. The prime editor protein is a fusion protein comprising SpCas9 nickase (nCas9) and Moloney murine leukemia virus (M-MLV) reverse transcriptase (RT). B. A plasmid vector expressing pegRNA under the U6 promoter and a puromycin resistance gene under the PGK promoter. The pegRNA consists of a spacer sequence specific to the target gene, a scaffold sequence required for complex formation with spCas9, a 3' extension sequence (labeled “3'Ext”) for editing, a linker sequence (labeled “L”), a tevopreQ1 sequence for enhancing RNA stability, and a terminator sequence (labeled “T”).

This step involves the construction of two essential plasmid vectors for the prime editing system (Figure 3). Each plasmid vector is composed of multiple components that need to be assembled to execute this protocol. Specifically, these include the prime editor gene, pegRNA sequence, and puromycin resistance gene for the selection of transfected cells. For this protocol, we used pCMV-PEmax-P2A-hMLH1dn (Addgene, plasmid number: 174828) as the PE4max expression vector. For next-generation PE7 [11], pCMV-PE7 (Addgene, plasmid number: 214812) should be purchased. In pluripotent stem cells, selecting a constitutive promoter that ensures robust gene expression is crucial. Previous studies have shown that the transcriptional activity of the CMV enhancer is significantly lower than that of the EF1A or CAG promoter in both human [14] and mouse [15] pluripotent stem cells. Therefore, we recommend replacing the CMV enhancer in pCMV-PEmax-P2A-hMLH1dn with the EF1A promoter to drive prime editor expression more effectively. Plasmids constructed using a similar strategy, including an EF1A promoter–driven prime editor and a pegRNA backbone vector with a puromycin resistance gene, are available from Addgene (plasmid numbers 184444 and 214085, respectively).

1. Amplify the insert fragment and backbone vector with PCR to obtain sufficient amounts for subsequent in-fusion cloning.

a. Prepare a 20 μL PCR reaction mixture (Table 3).

To amplify the EF1A promoter region, perform PCR using pLV[Exp]-EF1A>hCas9(ns):T2A:Puro (containing the EF1A promoter) as a template and EF1A_forward and EF1A_reverse primers.

To amplify the backbone, perform PCR using pCMV-PEmax-P2A-hMLH1dn as a template and Inverse_PEmax_forward and Inverse_PEmax_reverse primers.

**Table 3.** PCR reaction mixture components

Reagent	Final concentration	Volume
KOD One PCR master mix (dye-free 2×PCR master mix)	1×	10 μL
Forward primer (2 μM)	0.3 μM	3 μL
Reverse primer (2 μM)	0.3 μM	3 μL
Plasmid DNA (5 ng/µL)	1 ng/µL	4 μL
Total	n/a	μL

b. Perform two-step PCR using a thermal cycler under the temperature conditions provided in Table 4.

**Table 4.** Thermocycling conditions for PCR

Step	Temp. (°C)	Duration	No. of cycles
Initial denaturation	98	30 s	1
Denaturation	98	10 s	35
Extension	68	5 s/kb	
Hold	4	∞	-

c. Apply 1–2 μL of the PCR product to a 1.5% agarose gel containing a fluorescent nucleic acid stain (e.g., GelRed) and run electrophoresis at 100 V for approximately 15 min.

d. Visualize DNA bands on a transilluminator and verify that the PCR amplicon has migrated to the expected band size.

*Note: Consider using touchdown PCR or nested PCR when you need to increase DNA yield or specificity, such as when amplifying low-abundance targets or dealing with non-specific amplification.*

e. To remove the parental vector template, add 5–10 units of DpnI to 20 μL of the PCR post-reaction mixture and incubate at 37 °C for 10 min. Then, deactivate the enzyme by incubating at 80 °C for 10 min.

*Note: Plasmid DNA amplified in common* Escherichia coli *strains is methylated, while PCR products are typically unmethylated. DpnI specifically cleaves plasmids with methylated recognition sites. Therefore, linearized plasmids amplified by PCR using plasmid DNA as a template remain intact, while the template plasmid is degraded, preventing its carryover into transformation.*

f. Purify the target amplicon DNA from PCR products using a commercial DNA purification kit (e.g., NucleoSpin gel and PCR clean-up).

*Note 1: For single bands, direct purification of DNA from the post-PCR mixture is possible. If extra bands are present, indicating non-specific amplification, excise the desired-sized band from the gel after electrophoresis and extract DNA from the gel.*

*Note 2: Extensive washing twice with the wash buffer of the DNA purification kit has been shown to enhance the efficiency of subsequent in-fusion cloning.*

g. Quantify the extracted DNA concentration using a NanoDrop spectrophotometer at A260. Store the DNA at -20 °C for up to 6 months.

2. Mix the PCR products from step C1, the insert fragment, and the linearized vector, and perform cloning using the in-fusion snap assembly master mix.

a. Prepare a 5 μL in-fusion cloning reaction mixture. Mix the insert fragment and the linearized plasmid vector so that the approximate molar ratio is 2:1 (Table 5).

*Note: Carry out an in-fusion cloning reaction without the inclusion of an insert fragment to serve as a negative control for subsequent transformation experiments.*

**Table 5.** In-fusion cloning reaction mixture components

Reagent	Final concentration	Volume
In-fusion snap assembly master mix (5×)	1×	1 μL
Insert fragment	1–20 ng/μL	1 μL
linearized plasmid vector	5–20 ng/μL	1 μL
Nuclease-free water	n/a	To 5 μL
Total	n/a	5 μL

b. Carry out the in-fusion cloning reaction in a thermal cycler using the temperature conditions provided in Table 6.

**Table 6.** Thermocycling conditions for PCR

Step	Temp. (°C)	Duration
In-fusion reaction 1	37	15 min
In-fusion reaction 2	50	15 min
Hold	4	∞

c. Store the cloning reactions at -20 °C until ready to proceed with the transformation.

3. Transform *E. coli* with plasmids constructed using in-fusion cloning.

a. Combine 20 μL of competent cells with 0.5 μL of the in-fusion cloning reaction mixture and incubate on ice for 2 min.

Critical: To ensure a successful transformation, it is recommended that the amount of DNA is less than 2 ng for 20 μL of competent cells, and that the volume of TE buffer provided does not exceed 30% of the volume of the competent cells.

*Note: Use the in-fusion cloning mixture lacking the insert fragment from step C2a as a negative control for transformation.*

b. Heat shock the cells at 42 °C for 30 s and immediately transfer the cells to ice for 2 min.

c. Add 50 μL of SOC medium to the cells and shake at 37 °C for 30 min.

d. Spread the transformation mixture onto LB agar plates containing 40 µg/µL of ampicillin.

e. Incubate at 37 °C overnight (12–16 h).

*Note: If a high number of background colonies are observed on a negative control plate, this may indicate incomplete DpnI digestion and, consequently, reduced in-fusion cloning efficiency. To address this, increase the amount of DpnI added or extend the DpnI enzymatic reaction time.*

4. Perform colony PCR to select an *E. coli* colony that has been transformed with a plasmid vector into which the insert fragment has been inserted.

a. Prepare a 10 μL colony PCR reaction mixture (Table 7).

To amplify the EF1A promoter region, perform PCR using EF1A_forward and EF1A_reverse primers.

**Table 7.** Colony PCR reaction mixture components

Reagent	Final concentration	Volume
KOD One PCR master mix (dye-free 2× PCR master mix)	1×	10 μL
Forward primer (2 μM)	0.3 μM	3 μL
Reverse primer (2 μM)	0.3 μM	3 μL
Nuclease-free water	n/a	To 5 μL

b. Using a sterile pipette tip, transfer a small amount of each of 6–10 individual well-formed colonies to separate PCR tubes containing the prepared PCR mixture.

*Note: Use a permanent marker to number each colony on the plate to correlate the colony PCR results with the corresponding colonies.*

c. Perform two-step PCR using a thermal cycler under the temperature conditions in Table 8.

**Table 8.** Thermocycling conditions for PCR

Step	Temp. (°C)	Duration	No. of cycles
Initial denaturation	98	30 s	1
Denaturation	98	10 s	30
Extension	68	5 s/kb	
Hold	4	∞	-

d. Apply 1–2 μL of the PCR product to a 1.5% agarose gel containing a fluorescent nucleic acid stain (e.g., GelRed) and run electrophoresis at 100 V for approximately 15 min.

e. Visualize DNA bands on a transilluminator to identify colonies transformed with the successfully in-fusion-cloned plasmid vector.

5. Culture the transformed *E. coli* in liquid medium.

a. Using a pipette tip, touch the surface of 2–3 colonies confirmed to be positive for the insert fragment by colony PCR and inoculate them into 10 mL of BD DIFCO 2×YT (yeast extract tryptone broth) containing 40 µg/mL ampicillin in a 50 mL centrifuge tube.

*Note: To prevent cross-contamination, use a fresh pipette tip for each colony.*

b. Incubate at 37 °C for 16–20 h in a shaking incubator at 120–150 rpm.

*Note: To promote aerobic respiration in* E. coli, *slightly loosen the cap to improve aeration and tilt the tube slightly.*

c. Purify plasmids propagated by *E. coli* using commercial Miniprep kits (e.g., Plasmid Miniprep Plus Purification kit, BioElegen Technology).

Critical: Use an endotoxin-free plasmid purification kit to avoid unexpected cellular responses, such as cytotoxicity, during transfection.

d. Quantify the plasmid DNA concentration using a NanoDrop spectrophotometer at A260. Store the circular plasmids at -20 °C for up to 6 months.

Critical: Ensure the concentration of the purified plasmid DNA is at least 1,000 ng/µL to avoid negative impacts on cell viability and transfection efficiency, which can occur when excessive elution buffer is used.

6. Confirm the absence of DNA base substitutions around the in-fusion recombination site in the constructed plasmid vector with Sanger sequencing. Use EF1A_sequence_forward1, EF1A_sequence_forward2, and EF1A_sequence_reverse as sequencing primers.

7. Construct the pegRNA expression vector using PCR amplification and in-fusion cloning, following the same procedure as in steps C1–5. The backbone of the pegRNA expression vector, excluding sequence spanning from spacer to linker, is common regardless of the objective genomic locus or mutation. Clone this common sequence (e.g., tevopreQ1 and puromycin resistance gene) to create a backbone construct. Subsequently, clone the variable pegRNA sequences into the backbone construct using in-fusion cloning.

**Figure 4.**
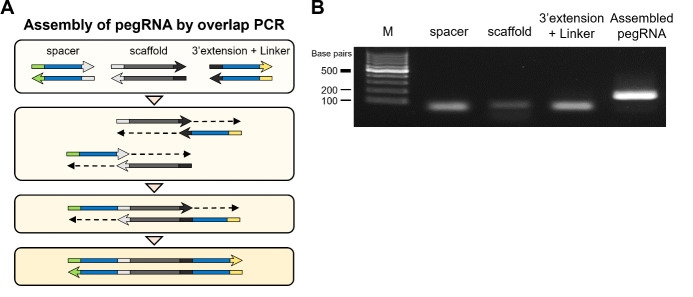
Assembling pegRNA using overlap PCR. A. The process of assembling pegRNA using overlap PCR. The homologous sequences of each component anneal to each other, initiating PCR and ultimately synthesizing the pegRNA spanning the entire region from the spacer to the linker sequences. B. Agarose gel electrophoresis image displaying bands corresponding to the PCR-amplified pegRNA components (spacer, spCas9 scaffold, and 3’extension + linker) and the assembled pegRNA. The target-specific band size is around 50 bp in the spacer, 80 bp in the spCas9 scaffold, 60 bp in the 3’extension + linker, and 170 bp in the assembled pegRNA. M: 100 bp DNA molecular weight marker.

*Note: While it is possible to directly insert three fragments (spacer, scaffold, and 3' extension + linker) into a vector using in-fusion cloning, we found that this approach often results in low cloning efficiency, typically less than 10%. To enhance efficiency, we recommend pre-assembling these fragments using overlap PCR (Figure 4). This method has consistently yielded cloning efficiencies of over 95% in our experiments. Although it requires additional synthesis costs, another option is to use a continuous DNA fragment spanning the entire region from the spacer to the linker, tailored to each designed pegRNA sequence.*

a. Amplify the insert fragment and backbone vector with PCR, following the same procedure as in steps C1a–d.

To amplify the spacer, spCas9 scaffold, 3’ extension + Linker sequence, and backbone, perform PCR using the corresponding primer pairs: Spacer_OPTN_forward/reverse, spCas9_scaffold_forward/reverse, 3’extension + Linker_OPTN_forward/reverse, and Inverse_U6-pegRNA_forward/reverse, respectively.

To remove the parental vector template, add 5–10 units of Dpn I to 20 μL of the PCR post-reaction mixture and incubate at 37 °C for 10 min, then deactivate the enzyme by incubating at 80 °C for 10 min.

b. Perform overlap PCR using the PCR-amplified spacer, spCas9 scaffold, and 3’extension + linker fragments as templates to obtain the desired pegRNA sequence as the PCR product. Use primers that amplify from both ends of the assembled pegRNA. Prepare a 20 μL PCR reaction mixture for each sample (Table 9).

To amplify the pegRNA sequence, perform PCR using Spacer_OPTN_forward and 3’extension + Linker_OPTN_reverse primers.

**Table 9.** Overlap PCR reaction mixture components

Reagent	Final concentration	Volume
KOD One PCR master mix (dye-free 2×PCR master mix)	1×	10 μL
Forward primer (2 μM)	0.3 μM	3 μL
Reverse primer (2 μM)	0.3 μM	3 μL
Post-PCR mixture (spacer)	n/a	1 μL
Post-PCR mixture (spCas9 scaffold)	n/a	2 μL
Post-PCR mixture (3’extension + Linker)	n/a	1 μL
Total	n/a	20 μL

c. Perform PCR following the same procedure as in steps C1b–g.

8. Following the same procedure as in steps C2–4, perform in-fusion cloning and transform the constructed plasmid vector into *E. coli*, followed by colony PCR.

To amplify the pegRNA sequence, perform colony PCR using Spacer_OPTN_forward and 3’extension + Linker_OPTN_reverse primers.

9. Following the same procedure as in steps C5–6, culture the transformed *E. coli*, purify the plasmid vector, and perform Sanger sequencing to confirm the correct sequence of the inserted fragment. Use the pegRNA_sequence_forward primer, which targets the U6 promoter sequence, to perform Sanger sequencing and analyze the pegRNA sequence downstream of the U6 promoter.

**D. Identification of highly efficient pegRNAs by Sanger sequencing analysis in HEK293T cells**

To enhance the success rate of generating genome-edited cell lines in cell types with low prime editing efficiency, such as iPS cells, it is crucial to select efficient pegRNAs in advance. Therefore, it is recommended to conduct preliminary experiments in cell types known to have high prime editing efficiency, like HEK293T cells, to narrow down efficient pegRNAs.

Specifically, the designed pegRNA and prime editor expression vectors are introduced into HEK293T cells, and the sequence of the target locus is analyzed. Sanger sequencing, which is a cost-effective and simple method, is used for sequence analysis. By comparing the analysis results using wave analysis software [e.g., ICE (Inference of CRISPR Edits) analysis], an approximate estimation of editing efficiency can be made based on the difference in the base sequence from the wild type.

While it is possible to skip this preliminary experiment and start experiments with iPS cells immediately, due to the low editing efficiency of iPS cells, it is recommended to perform more sensitive amplicon sequencing, although it is more costly. Amplicon sequencing involves PCR amplification of the target region followed by next-generation sequencing, allowing for more accurate evaluation of editing efficiency.

1. Ensure the health and viability of HEK293T cell cultures through routine maintenance.

a. Maintain HEK293T cells in DMEM supplemented with 10% FBS and 1% penicillin-streptomycin. Typically, 2 mL of medium is used per well of a 6-well plate. Incubate cells at 37 °C in a humidified atmosphere containing 5% CO_2_.

*Note: Aim for a confluency of 70%–90% with a cell density of approximately 2 × 10^5^–5 × 10^5^ cells/cm^2^.*

b. Observe cells daily under a phase-contrast microscope and change the medium every 2 days.

c. When cells reach 70%–90% confluence, wash the culture plate with 1 mL of DPBS per well of a 6-well plate and treat with 1 mL of 0.05% trypsin/EDTA at 37 °C for approximately 2–3 min. Neutralize the trypsin by adding 2 mL of DMEM (+10% FBS). Subsequently, transfer the cell suspension to a 15 mL centrifuge tube. After centrifuging at 200× *g* for 3 min, remove the supernatant. Gently resuspend the cells, avoiding bubbles, and subculture at a ratio of 1:100 to 1:50.

**Figure 5.**
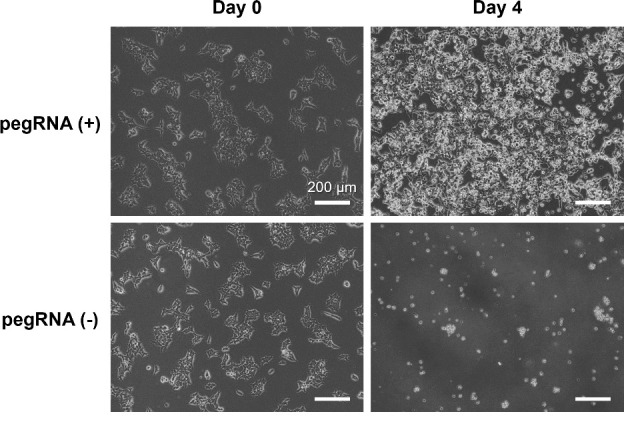
Representative image of HEK293T cells after being transfected and selected with puromycin. HEK293T cells were transfected with pegRNA and PEmax expression vectors on day 0 and cultured in a medium containing 5 μg/mL puromycin from days 1 to 4. The puromycin resistance gene in the pegRNA expression vector allowed for the selection of only the transfected cells by day 4. Scale bar indicates 200 μm.

2. Co-transfect HEK293T cells with pegRNA and PEmax expression vectors to introduce an SNV. Subsequently, perform puromycin selection to enrich for transfected cells (Figure 5).

*Note: To account for potential pipetting errors, prepare 1.2 times the required amount of all transfection reagents.*

a. Seed a suspension of viable HEK293T cells at a density of 2.0 × 10^4^ cells per well in a 48-well plate.

b. The following day, when the cells reach 20%–30% confluency, perform co-transfection with pegRNA and PEmax expression vectors. Replace the medium 1 h before transfection.

c. Per each well of a 48-well plate, add 125 ng each of pegRNA and PEmax expression vectors to a sterile 0.5 mL microcentrifuge tube containing 15 μL of DMEM. Set up a negative control with only PEmax expression vector.

d. Per each well of a 48-well plate, add 0.75 μL of PolyJet transfection reagent to a sterile 0.5 mL microcentrifuge tube containing 15 μL of DMEM.

e. Per each well of a 48-well plate, mix 15 μL of plasmid DNA/DMEM and 15 μL of PolyJet/DMEM.

f. Incubate at 20–25 °C for 10 min to allow DNA–polymer complex formation.

*Note: Never keep the PolyJet/DNA complex longer than 20 min.*

g. Per each well of a 48-well plate, add 30 μL of the DNA/PolyJet mixture and gently swirl the plate to ensure even distribution (day 0).

h. Replace the medium with fresh DMEM (+10% FBS) containing 5 µg/mL puromycin 24 h after transfection to initiate selection (day 1).

i. Change the medium daily. On day 4, switch to a puromycin-free medium.

*Note 1: To minimize cell detachment, gently change the medium by tilting the plate and allowing the medium to flow slowly down the wall.*

*Note 2: Non-transfected cells should begin detaching from the plate bottom and dying 24 h post-puromycin treatment. By day 4, there should be very few viable cells remaining in the negative control wells (see Troubleshooting).*

**Figure 6.**
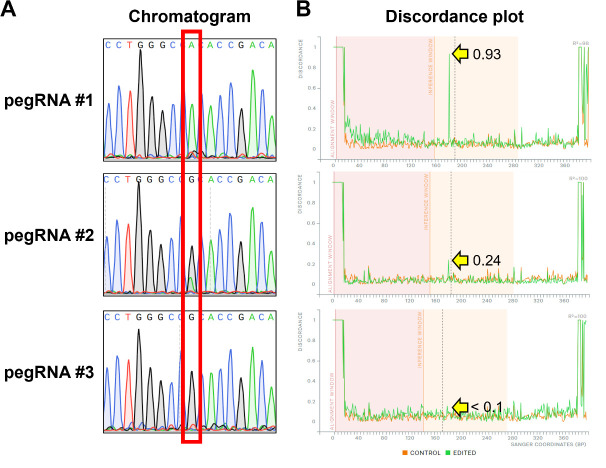
Analysis of bulk Sanger sequencing data from prime-edited HEK293T cells. Three pegRNAs with varying editing efficiencies (#1: high; #2: medium; #3: low) were used to prime edit a target gene in HEK293T cells, followed by bulk Sanger sequencing. Each pegRNA was designed to introduce a G-to-A missense mutation at chr9:129,098,086 of the human GRCh38 genome. A. Bulk Sanger sequencing analysis: the target editing site is indicated by the red box in the chromatogram. The editing efficiency of each pegRNA is estimated by comparing the peak height of the edited adenine base at the target site. A higher peak indicates a higher editing efficiency. B. ICE (inference of CRISPR edits) analysis: the discordance plot generated by the ICE software illustrates the base-by-base alignment between the wild-type (control; orange lines) and edited samples (green lines). It quantifies the degree of mismatch between the edited sample and the reference sequence derived from the control trace file. The degree of mismatch at the target locus after prime editing is indicated by the yellow arrow. The y-axis represents the level of discordance at each base, with higher values indicating greater editing efficiency. The vertical black lines represent the DNA cleavage sites introduced by each unique spacer sequence.

3. Compare the sequences of the target gene locus between wild-type and prime-edited cells to approximate the editing efficiency of each designed pegRNA (Figure 6).

a. As in steps A3–5, extract genomic DNA from the cells, perform PCR on the target gene locus, and confirm the sequence with Sanger sequencing.

b. Input the spacer sequence into the ICE analysis tool (https://ice.synthego.com/#/) and upload both the control file (unedited sample) and experiment file (edited sample) of the Sanger sequencing waveform data. The editing efficiency of each pegRNA is estimated by referring to the discordance plot and examining the divergence level at the target based on the discordance plot before and after prime editing.

**E. Co-transfect human iPS cells with pegRNA and PEmax expression vectors to introduce an SNV**

A variety of gene delivery methods have been developed to efficiently and safely introduce genetic material into cells, including physical methods (e.g., electroporation, magnetofection), biological methods (e.g., lentiviral transduction), and chemical methods (e.g., lipid- and polymer-based transfection).

Electroporation, a physical method, creates temporary pores in the cell membrane using an electric field, allowing for direct nucleic acid delivery into cells. Its versatility across different cell types makes it suitable for gene delivery into challenging cells such as iPS cells [16]. However, this method requires specialized equipment and has low throughput.

Chemical methods, on the other hand, use polymers or cationic lipids to form complexes with nucleic acids for cellular delivery. These positively charged delivery reagents interact with negatively charged nucleic acids to form complexes that adhere to the cell membrane through electrostatic interactions. Subsequently, these complexes are internalized into cells via endocytosis or phagocytosis. Compared to physical methods, chemical methods offer several advantages: they do not require specialized equipment, are virus-free, and can be performed using standard cell culture facilities. Additionally, the simplicity of the procedure enables simultaneous transfection under multiple conditions.

In this step, a polymer-based chemical transfection method is used to introduce genes into iPS cells. The concentration of the polymer-based transfection reagent has been optimized to minimize cytotoxicity in fragile iPS cells. Following transfection, non-transfected cells are eliminated by puromycin selection, and cells with the transgene are enriched. Single clones are then obtained by limiting dilution, a technique that ensures each clone originates from a single cell. Finally, clones with the desired gene mutation are identified by genotyping.

1. Ensure the health and undifferentiated state of human iPS cell cultures through routine maintenance.

a. Maintain iPS cells on 0.5 µg/cm^2^ of iMatrix-511-coated plates in StemFit medium supplemented with 1% penicillin-streptomycin. Typically, 1 mL of StemFit medium is used per well of a 12-well plate. Incubate cells at 37 °C in a humidified atmosphere containing 5% CO_2_. Observe cells daily under a phase-contrast microscope to monitor for any signs of differentiation, such as changes in colony morphology. Change the medium every 2 days or daily if the cells reach 40% confluence.

*Note: Aim for a confluency of 60%–80% with a cell density of approximately 5 × 10^5^–1 × 10^6^ cells/cm^2^.*

b. When the cells reach 60%–80% confluency, wash the culture plate with 1 mL of DPBS per well of a 12-well plate and treat with 500 μL of TrypLE Select at 37 °C for approximately 6–9 min. Observe the cells under a microscope until the cell boundaries become distinct and the cells round up. Then, remove the TrypLE Select and add 1 mL of StemFit medium containing 10 µM Y-27632 per well of a 12-well plate. Gently pipette the medium along the bottom of the well to easily collect the cells without damaging them. Subsequently, transfer the cell suspension to a 1.5 mL tube. After centrifuging at 200× *g* for 3 min, remove the supernatant. Gently resuspend the cells with 1 mL of StemFit medium (+10 µM Y-27632), avoiding bubbles, and subculture with a cell density of 8.5 × 10^2^–3.5 × 10^3^ cells/cm^2^. The following day, confirm colony formation and replace the medium with Y-27632-free StemFit medium.

**Figure 7.**
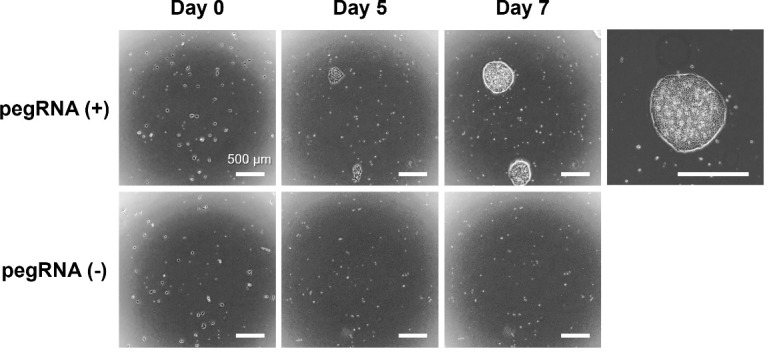
Representative image of human iPS cells after being transfected and selected with puromycin. The human iPS cell line 201B7 was transfected with pegRNA and PEmax expression vectors on day 0 and cultured in a medium containing 0.25 μg/mL puromycin from days 1 to 4. The puromycin resistance gene in the pegRNA expression vector allowed for the selection of only the transfected cells by day 4. Cell colonies of the transfected and selected cells reached a diameter of approximately 500 µm on days 7–10. Scale bar indicates 500 μm.

2. Co-transfect iPS cells with pegRNA and PEmax expression vectors to introduce an SNV. Subsequently, perform puromycin selection to enrich for transfected cells (Figure 7).

*Note: To account for potential pipetting errors, prepare 1.2 times the required amount of all transfection reagents.*

a. When the cells reach 60%–80% confluence and are actively proliferating, perform co-transfection with pegRNA and PEmax expression vectors. Detach the cells from the culture plate using the same procedure as in step E1 and transfer 1× 10^5^ cells to a 0.5 mL tube. Set up cells for a negative control using only the PEmax expression vector. After preparing the transfection reagent as detailed in steps E2b–e, centrifuge the cells at 200× *g* for 3 min to pellet them.

b. To a sterile 0.5 mL microcentrifuge tube containing 10 μL of DMEM, add 100 ng of pegRNA and 100 ng of PEmax expression vectors per 1 × 10^5^ cells. Set up a negative control with only PEmax expression vector.

c. Per 1 × 10^5^ cells, add 0.8 μL of PolyJet transfection reagent to a sterile 0.5 mL microcentrifuge tube containing 10 μL of DMEM.

d. Per 1 × 10^5^ cells, mix 10 μL of plasmid DNA/DMEM and 10 μL of PolyJet/DMEM.

e. Incubate at 20–25 °C for 10 min to allow DNA–polymer complex formation.

*Note: Never keep the PolyJet/DNA complex longer than 20 min. Centrifuge the cells to form a cell pellet immediately before the end of this incubation.*

f. Perform a 5-fold dilution of the PolyJet-DNA mixture by adding 20–80 μL of DMEM per 1 × 10^5^ cells.

*Note: This is the optimal dilution ratio of the PolyJet–DNA mixture to suppress cytotoxicity in the human iPS cell line 201B7. Since the optimal dilution ratio varies depending on the cell line, it is necessary to optimize the ratio for each cell line as follows: The PolyJet-DNA mixture is prepared according to the manufacturer’s protocol and then serially diluted from the undiluted solution to a 5-fold dilution. Transfection is performed on the iPS cell line at each dilution. The maximum concentration of the transfection reagent that allows for >90% cell viability the following day is determined to be the optimal one.*

g. After removing the supernatant, add 100 μL of the diluted PolyJet–DNA mixture per 1 × 10^5^ cells and gently resuspend the cells by pipetting.

h. Incubate the cells at 37 °C for 20 min.

i. Add 10–20 μL of cell suspension per well (approximately 5 × 10^3^–1 × 10^4^ cells) to 12-well plates pre-coated with 0.5 µg/cm^2^ of iMatrix-511 and containing 1 mL of StemFit medium supplemented with 10 μM Y-27632 (day 0). Pipette gently with a 1 mL pipette to evenly distribute the cells throughout the well.

j. Replace the medium with 1 mL of fresh StemFit medium containing 0.25 μg/mL puromycin and 10 μM Y-27632 24 h after transfection to initiate selection (day 1).

*Note: The optimal concentration of puromycin varies depending on the iPS cell line, so it is necessary to optimize it for your specific cell line as follows: Transfect cells with or without a puromycin expression vector, and then treat them with 0.1–0.5 µg/mL of puromycin. The optimal concentration of puromycin is considered to be the concentration at which no viable cells remain in the negative control, but viable colonies are observed in the transfected wells.*

k. Change the medium daily. On day 4, switch to a puromycin-free StemFit medium (+10 μM Y-27632).

*Note 1: To minimize cell detachment, gently change the medium by tilting the plate and allowing the medium to flow slowly down the wall.*

*Note 2: Non-transfected cells should begin detaching from the plate bottom and dying 24 h after puromycin treatment. By day 4, there should be very few viable cells remaining in the negative control wells (see Troubleshooting).*

l. Change the medium with fresh StemFit medium every 1–2 days until the cell colonies reach a diameter of 500 μm or more (Figure 7).

*Note: In each well of the 12-well plate, it is expected that more than 20–30 cell colonies will be formed and that the total number of cells will be more than 2 × 10^4^–4 × 10^4^. However, at this stage, the transfected cells have only been selected, so it is not certain that each colony is a genetically identical single clone.*

3. Detach the cell colonies formed in step E2 and seed them into a 96-well plate by limiting dilution to obtain single clones.

a. Following step E1, trypsinize the cells using TrypLE Select. Prepare a single-cell suspension at a concentration of 10 cells/mL in a reservoir. Mix the cell suspension well to homogenize it using a large pipette and seed 100 μL aliquots into a 96-well plate coated with 0.5 μg/cm^2^ of iMatrix-511 to achieve a cell density of approximately 1 cell per well.

b. Observe the wells daily and record the wells in which colonies have formed. As a backup, it is also possible to use a part of the cell for expansion culture.

*Note 1: If you observe two or more colonies in a single well, or if a colony is significantly larger than others (more than twice the diameter), it may not be a single clone. However, since it is possible that the desired mutation has been introduced, as will be confirmed by the subsequent sequencing analysis, do not exclude these wells but proceed with the following steps. After sequencing analysis, if necessary, perform limiting dilution again to obtain single clones.*

*Note 2: Ensure that 200 μL of media is added per well to minimize the meniscus effect and allow for clear observation of the edges of the wells. Expect to see colony formation in at least 20%–40% of the wells.*

4. Culture until colonies reach a diameter of 400–600 μm. Re-disperse the formed colonies and seed them into 96- or 48-well plates for expansion culturing.

*Note: At this step, it is also possible to use half of the cells for direct PCR in section F.*

5. When the cells reach 60%–80% confluence, detach the cells and aliquot the cell suspension into 0.5 mL tubes for cell banking and genotyping. For cell banking, cell pellets are resuspended in 200 μL of STEM-CELLBANKER and stored at -80 °C. For genotyping, cell pellets are stored at -30 °C. As a backup, it is also possible to use a part of the cell for expansion culture.

*Note 1: For genotyping, a cell pellet equivalent to 1/2 to 1/20 of the cells from a single well of a 96-well plate is sufficient.*

*Note 2: Ensure that each clone’s cell bank and genotyping frozen pellet are numbered consistently.*

**F. Genotyping of single clones with direct PCR**

Establishing and identifying a specific cell line with the desired genetic mutation from numerous candidate cell lines is a time-consuming and laborious process. This paper introduces an improved method for streamlining this process: rapid detection of gene mutations using direct PCR with cell pellets. This method eliminates the need for the conventional, time-consuming process of genomic DNA purification, allowing for faster identification of the target cell line. Specifically, direct PCR and Sanger sequencing are used to amplify the target gene region and directly confirm the presence or absence of the desired mutation.

1. Perform direct PCR on the frozen cell pellet from step E5 to amplify the target locus for genotyping.

a. Prepare a 30 μL PCR reaction mixture for each sample (Table 10). Prepare a master mix of 1.1 to 1.2 times the total volume of PCR mix required and dispense 24 μL into each PCR tube.

To amplify the region of the *OPTN* p.(Asn51Thr) missense mutation, perform PCR using OPTN_genotyping_forward and OPTN_genotyping_reverse primers.

**Table 10.** PCR reaction mixture components

Reagent	Final concentration	Volume
KOD One PCR master mix (dye-free 2×PCR master mix)	1×	15 μL
Forward primer (2 μM)	0.3 μM	4.5 μL
Reverse primer (2 μM)	0.3 μM	4.5 μL
Sample	n/a	1 μL
Nuclease-free water	n/a	To 30 μL
Total	n/a	30 μL

b. Add 10 μL of DPBS to the cell pellet from step E5 and mix well by pipetting or pulse vortexing.

c. Add 1 μL of each cell sample to the PCR reaction mixture, mix by inverting, and then spin down.

d. Perform PCR according to the procedure in steps A1–5, confirm the amplification product by electrophoresis, purify it, and perform Sanger sequencing. By doing so, the genotype of each single-cell line can be identified.

*Note: When the PCR product yield is low, increasing the cycle number to approximately 40 can help amplify the product. If this is insufficient, re-PCR using the primary PCR product as a template can be an effective strategy. However, it is important to note that increasing the cycle number also increases the risk of amplification errors, potentially increasing the risk of misinterpreting sequence analysis. Alternatively, although additional steps are required, the efficiency of PCR amplification can be improved by using purified genomic DNA derived from cell pellets as a PCR template.*

**Figure 8.**
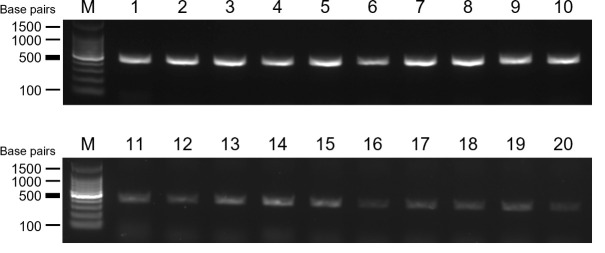
Direct PCR amplification of the target gene from cell pellets. Agarose gel electrophoresis image displaying bands corresponding to the target gene (*OPTN*) amplified by direct PCR with cell pellets suspended in DPBS. Samples 1–10 exhibited successful amplification, while samples 11–20 showed insufficient amplification, suggesting the need for additional PCR reactions. The target-specific band size is around 400 bp. M: 100 bp DNA molecular weight marker: lanes 1–20: samples 1–20.

2. Subsequently, expand the cell line with the desired genetic mutation. To enhance the clone purity of the target mutant, serial dilution can be repeated as needed. At least five aliquots of the obtained clones should be prepared and stored in a -80 °C ultra-low temperature freezer for up to 6 months and in liquid nitrogen for long-term storage. This minimizes the reduction in cell viability upon thawing, enabling repeated use in experiments.

## Data analysis

Accurate analysis with Sanger sequencing is essential for the selection of efficient pegRNAs and precise identification of clones with target gene mutations. As described in section A, it is crucial to first confirm that PCR and Sanger sequencing can be successfully performed on a wild-type sample at the target locus. The obtained Sanger sequencing data consists of two parts: FASTA sequence data and waveform data. In particular, waveform data are crucial for evaluating the quality of the sequencing reaction. When analyzing waveform data, it is necessary to carefully check whether the waveform is stable and of high quality throughout the sequence, whether there are multiple overlapping peaks, and whether there are any regions with low-quality values. Since the first and last 50 bases of the read tend to have lower quality, the waveform in the middle region should be evaluated more closely. If abnormalities are observed in the waveform data, it may indicate non-specific amplification in the PCR reaction or low quality of the purified DNA.

## Validation of protocol

Using this protocol, we introduced the missense mutation *OPTN* p.(Asn51Thr) into wild-type human iPS cells and obtained the desired mutant clones with an efficiency of 20%. In an independent experiment targeting the same locus, we achieved 16.7% efficiency. Furthermore, we introduced single nucleotide substitutions at two additional loci, chr9: 129,098,086 (G>A) and chr9: 129,101,921 (G>A), obtaining the desired clones with efficiencies of 2.3% and 5.3%, respectively. These results confirm the efficacy of this protocol for introducing SNVs into human iPS cells.

## General notes and troubleshooting

**General notes**

One limitation of this protocol is the difficulty in selecting highly efficient pegRNAs for human iPS cells in silico.

While deep learning based on experimental data enables the in silico prediction of highly efficient pegRNAs in HEK293T cells, this approach has not been widely applied to human iPS cells, and further data are needed. Currently, preliminary experiments using HEK293T cells or amplicon sequencing of edited iPS cells are considered options for pegRNA selection.

**Troubleshooting**

Problem 1: Low yield or no amplification of the pegRNA sequence in overlap PCR.

Possible cause: The annealing temperature may be too low due to the base composition and length of the overlap regions in each component.

Solution: Extend the length of the overlap region by an additional 5 base pairs or more.

Problem 2: Cells in the negative control are surviving and proliferating during puromycin selection.

Possible cause: The sensitivity to puromycin can vary depending on the cell line and cell density. Cells at higher densities may be less susceptible to puromycin.

Solution: To ensure the effectiveness of puromycin selection, it is crucial to verify its storage conditions and consider the characteristics of the cell line being used. Puromycin should be stored at −20 °C in the dark to maintain its activity. If the cell line exhibits resistance to puromycin, alternative antibiotics like hygromycin B or G418 can be explored. Additionally, optimizing puromycin concentration and cell density can enhance selection efficiency, especially if there are no underlying issues with the reagents or cell line. The optimal puromycin concentration for iPS cells is between 0.1 and 1 µg/mL, while for HEK293T cells, it ranges from 1 to 10 µg/mL.

Problem 3: No surviving cells are observed after puromycin selection.

Possible cause: The transfection efficiency may be too low, or the sensitivity to puromycin may vary depending on cell line and cell density. Cells with low viability may be more susceptible to puromycin.

Solution: Use a reporter expression vector, such as EGFP, to determine the transfection efficiency of your cell line. If no transfected cells are observed, consider increasing the concentration of the transfection reagent or switching to a different transfection reagent. Alternatively, start selection with half the puromycin concentration specified in the protocol. Additionally, gentle pipetting and other handling procedures during passaging can help maintain cell viability and make them less susceptible to puromycin.

Problem 4: The target gene sequence is not amplified by direct PCR.

Possible causes: Low primer specificity or the genomic sequence may be GC-rich or AT-rich, making it difficult to amplify.

Solution: Raise the annealing temperature, redesign the primers for higher specificity, or use a PCR enzyme designed for amplifying GC-rich or AT-rich sequences, such as PrimeStar GXL DNA polymerase (Takara).
